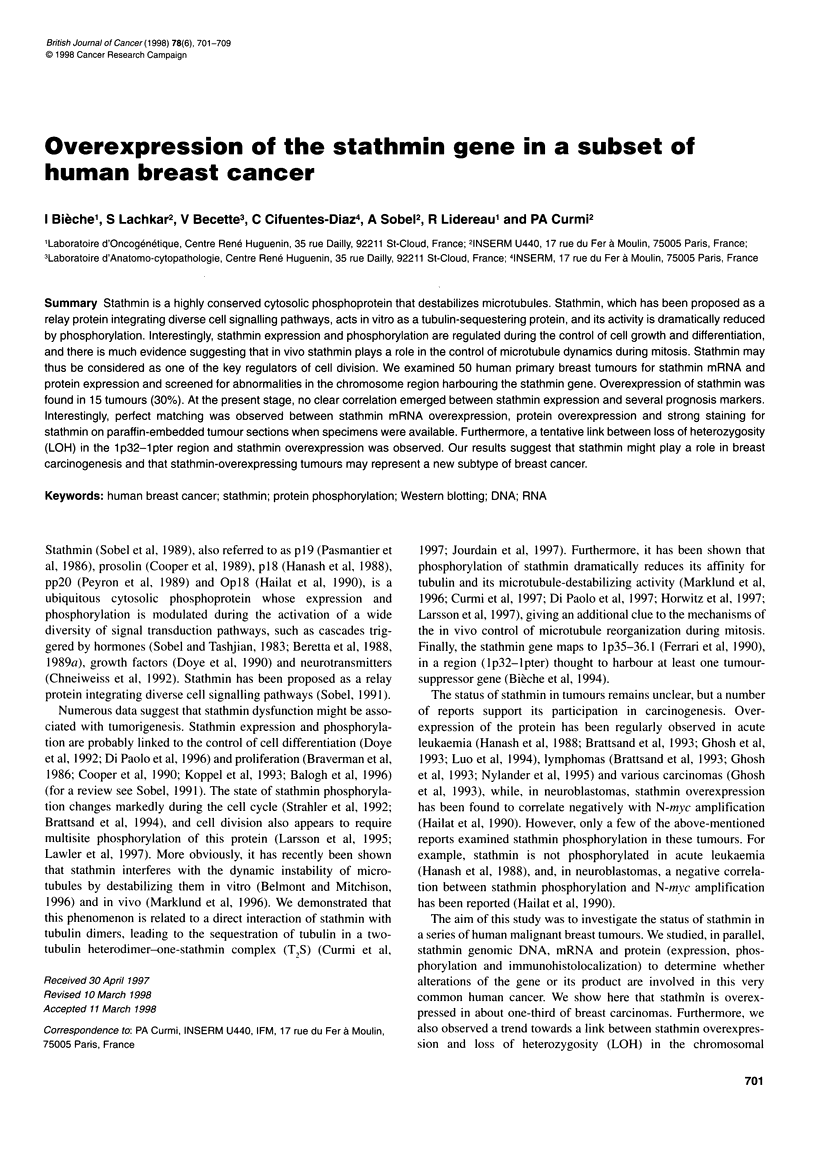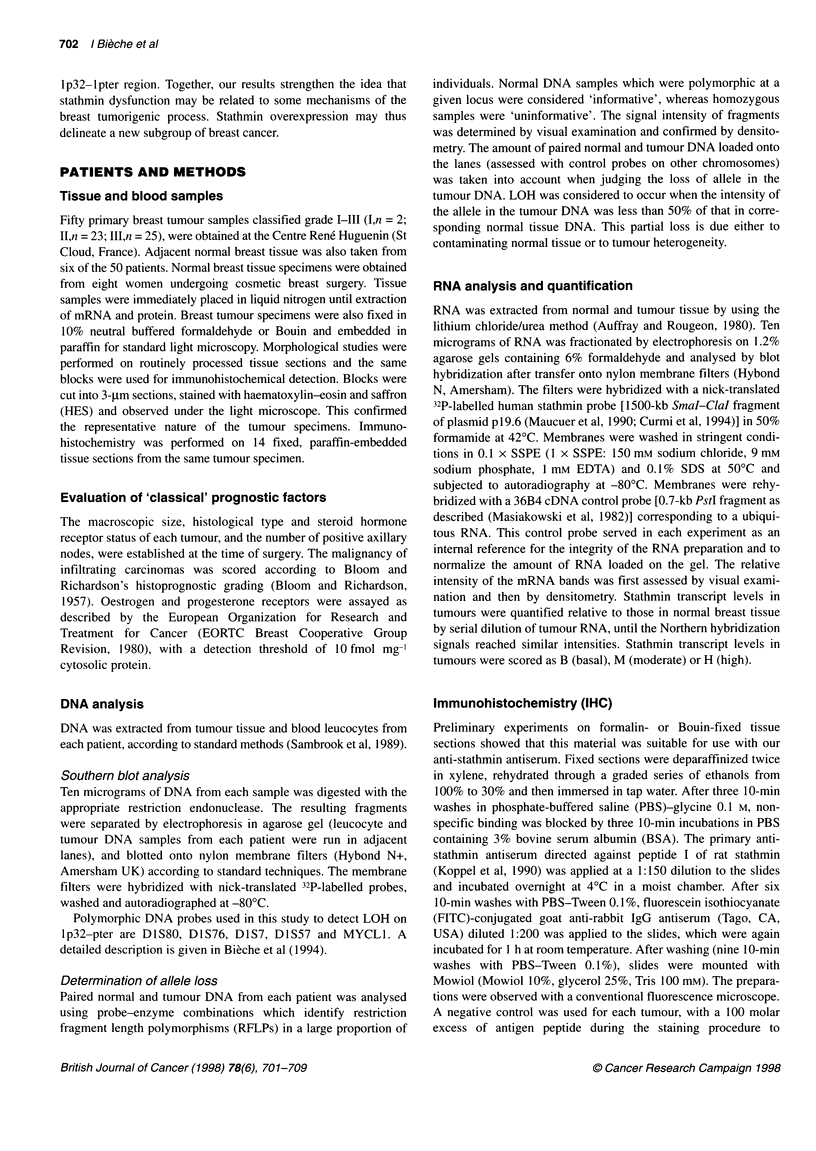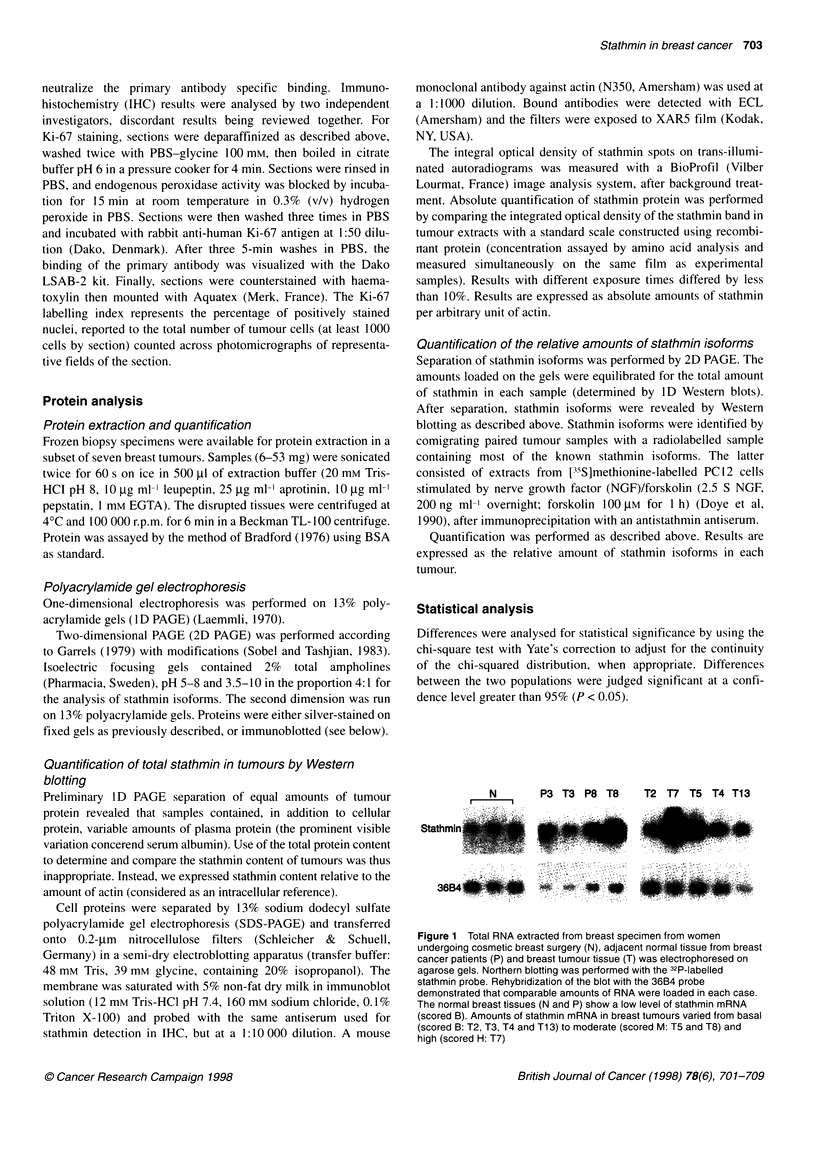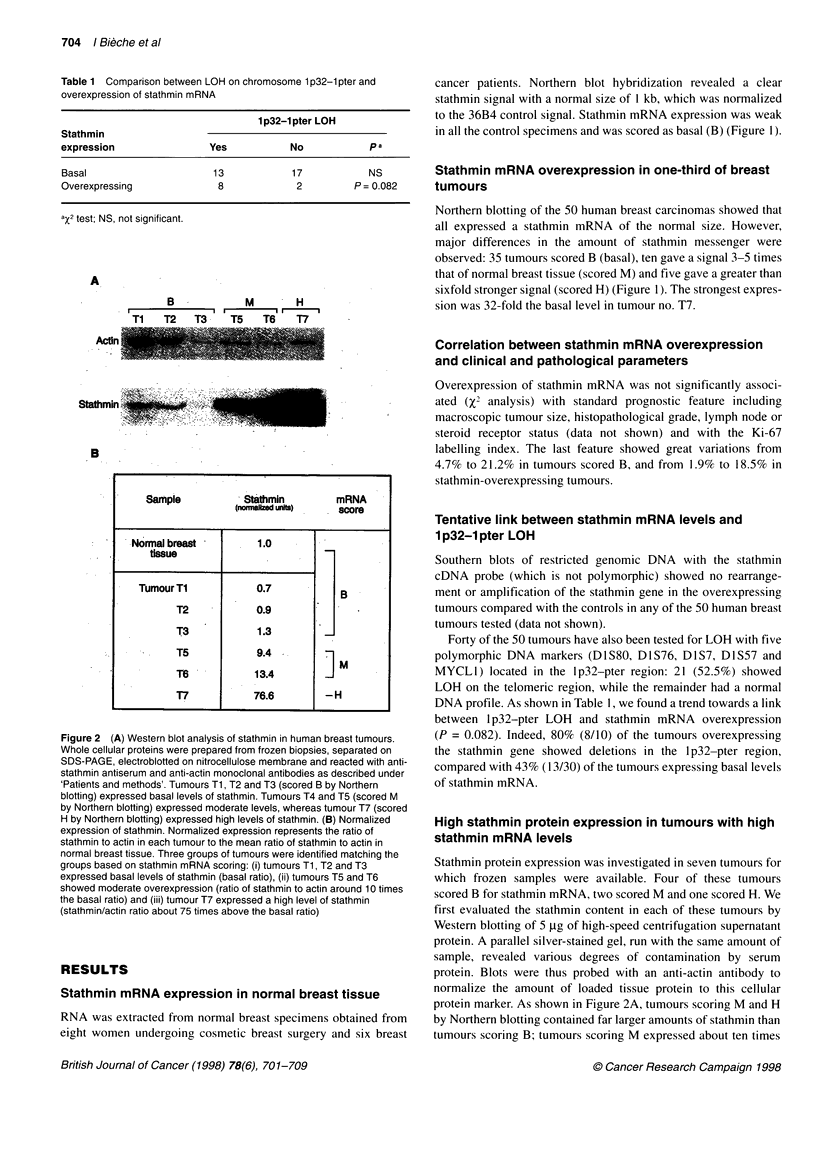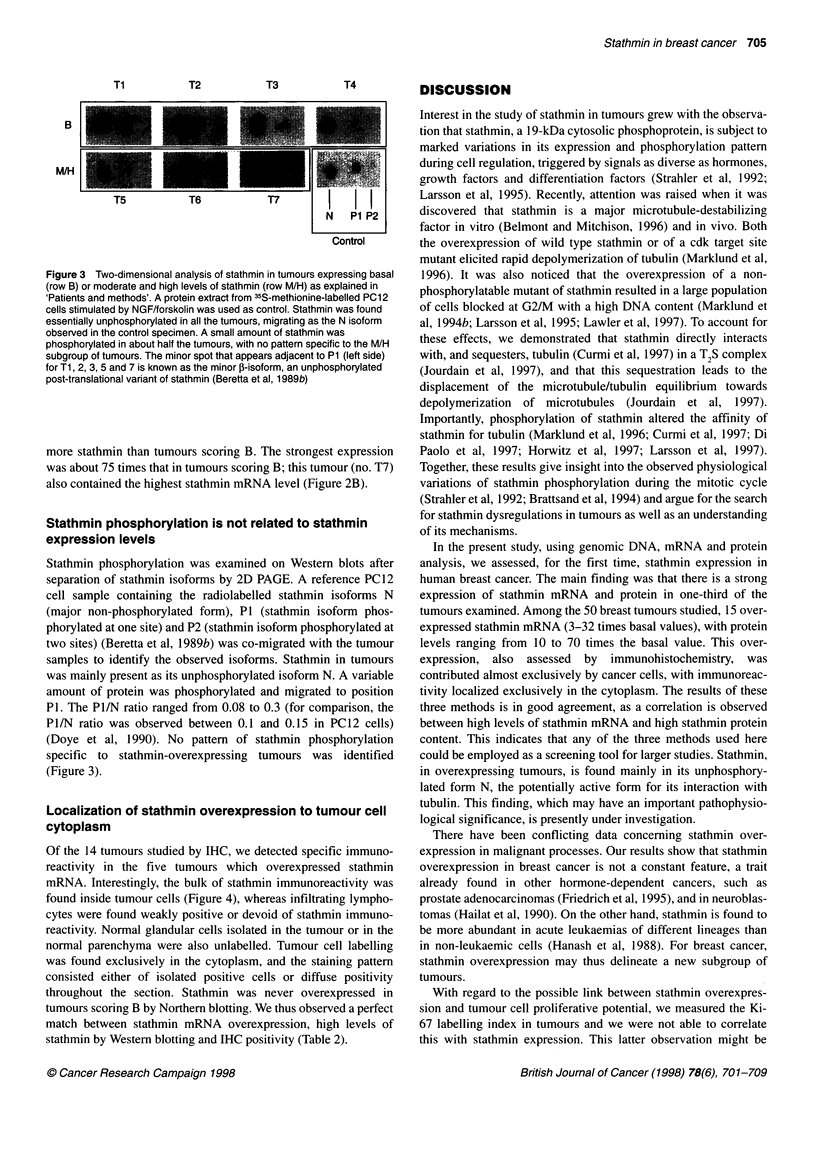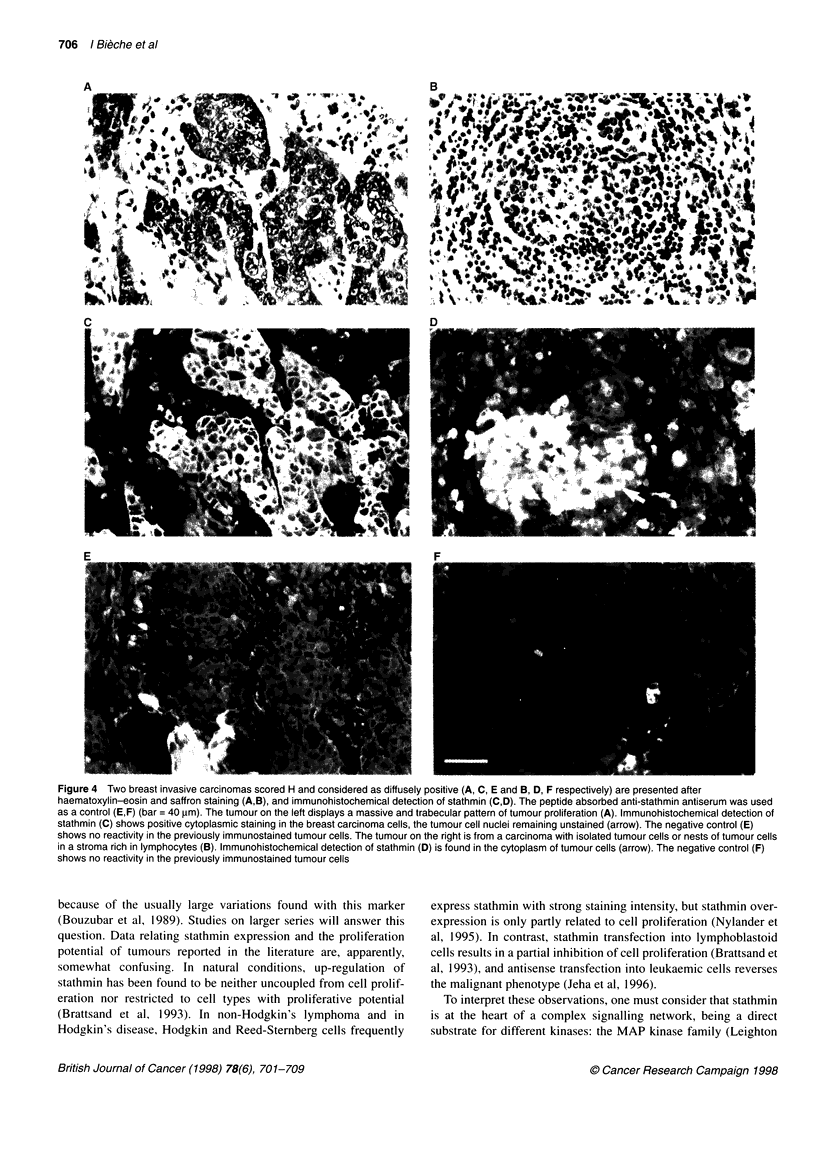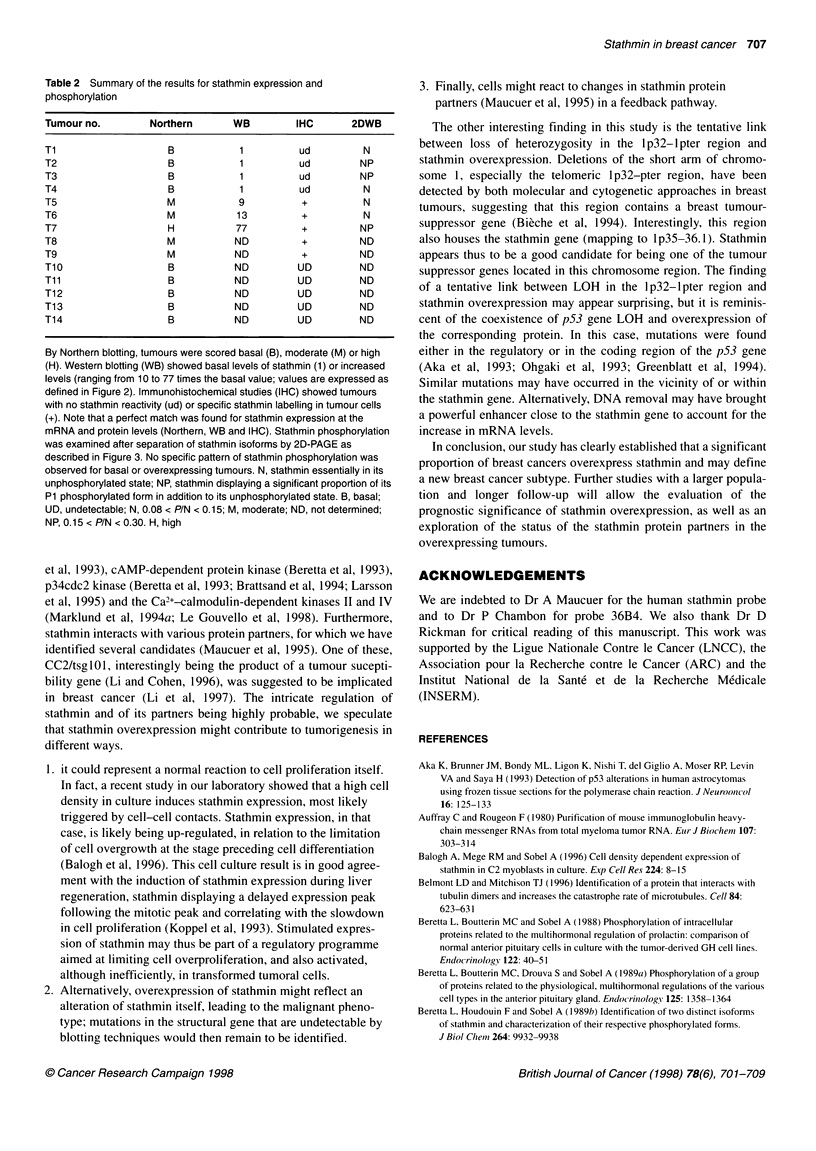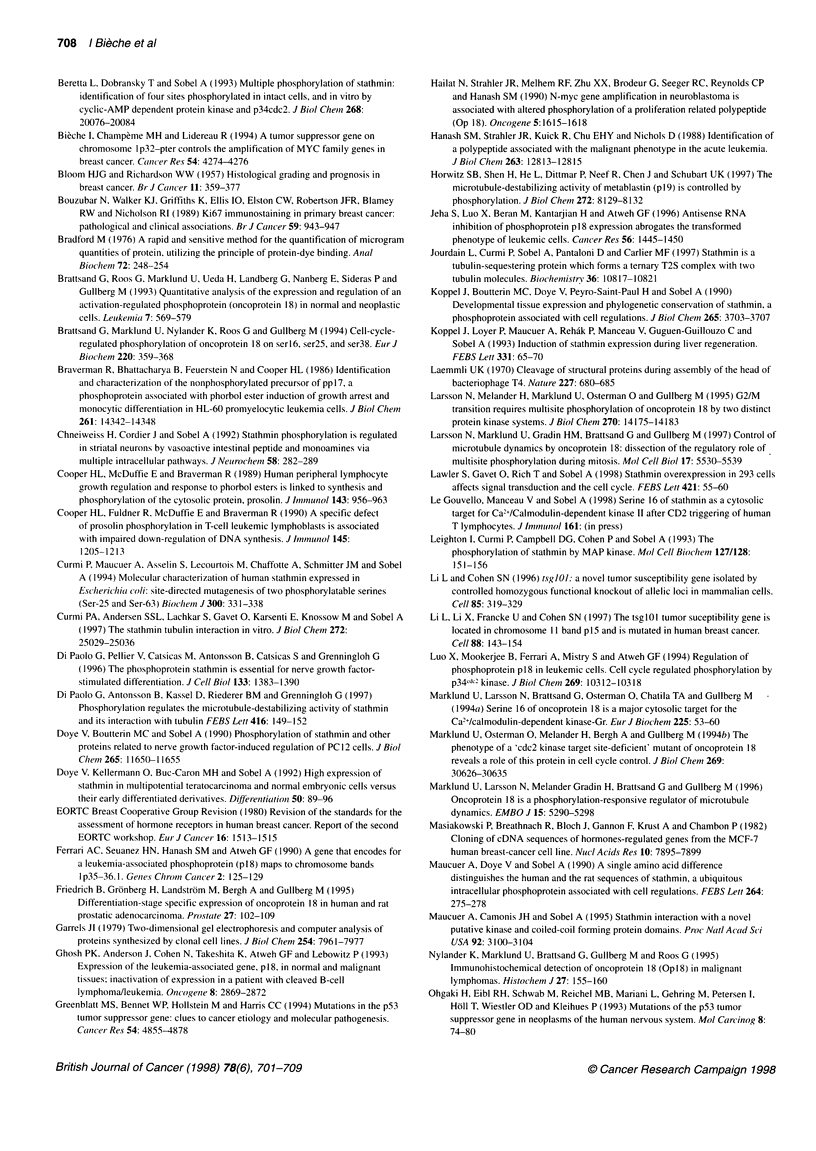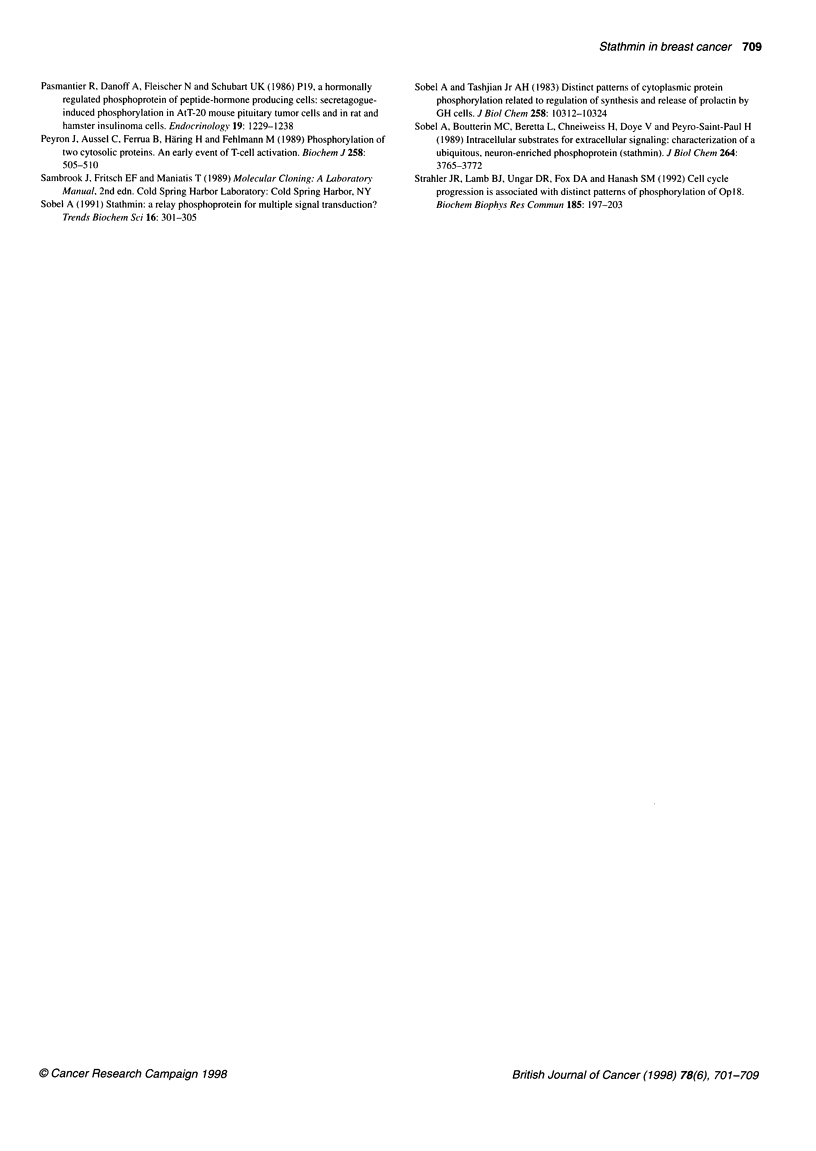# Overexpression of the stathmin gene in a subset of human breast cancer.

**DOI:** 10.1038/bjc.1998.565

**Published:** 1998-09

**Authors:** I. Bièche, S. Lachkar, V. Becette, C. Cifuentes-Diaz, A. Sobel, R. Lidereau, P. A. Curmi

**Affiliations:** Laboratoire d'Oncologénétique, Centre René Huguenin, St-Cloud, France.

## Abstract

**Images:**


British Journal of Cancer (1998) 78(6), 701–709
© 1998 Cancer Research Campaign

### Overexpression of the stathmin gene in a subset of human breast cancer

I Bièche[1], S Lachkar[2], V Becette[3], C Cifuentes-Diaz[4], A Sobel[2], R Lidereau[1] and PA Curmi[2]

[1]Laboratoire d'Oncogénétique, Centre René Huguenin, 35 rue Dailly, 92211 St-Cloud, France; [2]INSERM U440, 17 rue du Fer à Moulin, 75005 Paris, France; [3]Laboratoire d'Anatomo-cytopathologie, Centre René Huguenin, 35 rue Dailly, 92211 St-Cloud, France; [4]INSERM, 17 rue du Fer à Moulin, 75005 Paris, France

**Summary** Stathmin is a highly conserved cytosolic phosphoprotein that destabilizes microtubules. Stathmin, which has been proposed as a relay protein integrating diverse cell signalling pathways, acts in vitro as a tubulin-sequestering protein, and its activity is dramatically reduced by phosphorylation. Interestingly, stathmin expression and phosphorylation are regulated during the control of cell growth and differentiation, and there is much evidence suggesting that in vivo stathmin plays a role in the control of microtubule dynamics during mitosis. Stathmin may thus be considered as one of the key regulators of cell division. We examined 50 human primary breast tumours for stathmin mRNA and protein expression and screened for abnormalities in the chromosome region harbouring the stathmin gene. Overexpression of stathmin was found in 15 tumours (30%). At the present stage, no clear correlation emerged between stathmin expression and several prognosis markers. Interestingly, perfect matching was observed between stathmin mRNA overexpression, protein overexpression and strong staining for stathmin on paraffin-embedded tumour sections when specimens were available. Furthermore, a tentative link between loss of heterozygosity (LOH) in the 1p32–1pter region and stathmin overexpression was observed. Our results suggest that stathmin might play a role in breast carcinogenesis and that stathmin-overexpressing tumours may represent a new subtype of breast cancer.

**Keywords:** human breast cancer; stathmin; protein phosphorylation; Western blotting; DNA; RNA

Stathmin (Sobel et al, 1989), also referred to as p19 (Pasmantier et al, 1986), prosolin (Cooper et al, 1989), p18 (Hanash et al, 1988), pp20 (Peyron et al, 1989) and Op18 (Hailat et al, 1990), is a ubiquitous cytosolic phosphoprotein whose expression and phosphorylation is modulated during the activation of a wide diversity of signal transduction pathways, such as cascades triggered by hormones (Sobel and Tashjian, 1983; Beretta et al, 1988, 1989*a*), growth factors (Doye et al, 1990) and neurotransmitters (Chneiweiss et al, 1992). Stathmin has been proposed as a relay protein integrating diverse cell signalling pathways (Sobel, 1991).

Numerous data suggest that stathmin dysfunction might be associated with tumorigenesis. Stathmin expression and phosphorylation are probably linked to the control of cell differentiation (Doye et al, 1992; Di Paolo et al, 1996) and proliferation (Braverman et al, 1986; Cooper et al, 1990; Koppel et al, 1993; Balogh et al, 1996) (for a review see Sobel, 1991). The state of stathmin phosphorylation changes markedly during the cell cycle (Strahler et al, 1992; Brattsand et al, 1994), and cell division also appears to require multisite phosphorylation of this protein (Larsson et al, 1995; Lawler et al, 1997). More obviously, it has recently been shown that stathmin interferes with the dynamic instability of microtubules by destabilizing them in vitro (Belmont and Mitchison, 1996) and in vivo (Marklund et al, 1996). We demonstrated that this phenomenon is related to a direct interaction of stathmin with tubulin dimers, leading to the sequestration of tubulin in a two-tubulin heterodimer–one-stathmin complex ($T_2S$) (Curmi et al, 1997; Jourdain et al, 1997). Furthermore, it has been shown that phosphorylation of stathmin dramatically reduces its affinity for tubulin and its microtubule-destabilizing activity (Marklund et al, 1996; Curmi et al, 1997; Di Paolo et al, 1997; Horwitz et al, 1997; Larsson et al, 1997), giving an additional clue to the mechanisms of the in vivo control of microtubule reorganization during mitosis. Finally, the stathmin gene maps to 1p35–36.1 (Ferrari et al, 1990), in a region (1p32–1pter) thought to harbour at least one tumour-suppressor gene (Bièche et al, 1994).

The status of stathmin in tumours remains unclear, but a number of reports support its participation in carcinogenesis. Overexpression of the protein has been regularly observed in acute leukaemia (Hanash et al, 1988; Brattsand et al, 1993; Ghosh et al, 1993; Luo et al, 1994), lymphomas (Brattsand et al, 1993; Ghosh et al, 1993; Nylander et al, 1995) and various carcinomas (Ghosh et al, 1993), while, in neuroblastomas, stathmin overexpression has been found to correlate negatively with N-*myc* amplification (Hailat et al, 1990). However, only a few of the above-mentioned reports examined stathmin phosphorylation in these tumours. For example, stathmin is not phosphorylated in acute leukaemia (Hanash et al, 1988), and, in neuroblastomas, a negative correlation between stathmin phosphorylation and N-*myc* amplification has been reported (Hailat et al, 1990).

The aim of this study was to investigate the status of stathmin in a series of human malignant breast tumours. We studied, in parallel, stathmin genomic DNA, mRNA and protein (expression, phosphorylation and immunohistolocalization) to determine whether alterations of the gene or its product are involved in this very common human cancer. We show here that stathmin is overexpressed in about one-third of breast carcinomas. Furthermore, we also observed a trend towards a link between stathmin overexpression and loss of heterozygosity (LOH) in the chromosomal

*Received 30 April 1997*
*Revised 10 March 1998*
*Accepted 11 March 1998*

*Correspondence to*: PA Curmi, INSERM U440, IFM, 17 rue du Fer à Moulin, 75005 Paris, France

1p32–1pter region. Together, our results strengthen the idea that stathmin dysfunction may be related to some mechanisms of the breast tumorigenic process. Stathmin overexpression may thus delineate a new subgroup of breast cancer.

### PATIENTS AND METHODS

### Tissue and blood samples

Fifty primary breast tumour samples classified grade I–III (I,$n$ = 2; II,$n$ = 23; III,$n$ = 25), were obtained at the Centre René Huguenin (St Cloud, France). Adjacent normal breast tissue was also taken from six of the 50 patients. Normal breast tissue specimens were obtained from eight women undergoing cosmetic breast surgery. Tissue samples were immediately placed in liquid nitrogen until extraction of mRNA and protein. Breast tumour specimens were also fixed in 10% neutral buffered formaldehyde or Bouin and embedded in paraffin for standard light microscopy. Morphological studies were performed on routinely processed tissue sections and the same blocks were used for immunohistochemical detection. Blocks were cut into 3-μm sections, stained with haematoxylin–eosin and saffron (HES) and observed under the light microscope. This confirmed the representative nature of the tumour specimens. Immunohistochemistry was performed on 14 fixed, paraffin-embedded tissue sections from the same tumour specimen.

### Evaluation of 'classical' prognostic factors

The macroscopic size, histological type and steroid hormone receptor status of each tumour, and the number of positive axillary nodes, were established at the time of surgery. The malignancy of infiltrating carcinomas was scored according to Bloom and Richardson's histoprognostic grading (Bloom and Richardson, 1957). Oestrogen and progesterone receptors were assayed as described by the European Organization for Research and Treatment for Cancer (EORTC Breast Cooperative Group Revision, 1980), with a detection threshold of 10 fmol $mg^{-1}$ cytosolic protein.

### DNA analysis

DNA was extracted from tumour tissue and blood leucocytes from each patient, according to standard methods (Sambrook et al, 1989).

#### *Southern blot analysis*

Ten micrograms of DNA from each sample was digested with the appropriate restriction endonuclease. The resulting fragments were separated by electrophoresis in agarose gel (leucocyte and tumour DNA samples from each patient were run in adjacent lanes), and blotted onto nylon membrane filters (Hybond N+, Amersham UK) according to standard techniques. The membrane filters were hybridized with nick-translated $^{32}$P-labelled probes, washed and autoradiographed at –80°C.

Polymorphic DNA probes used in this study to detect LOH on 1p32–pter are D1S80, D1S76, D1S7, D1S57 and MYCL1. A detailed description is given in Bièche et al (1994).

#### *Determination of allele loss*

Paired normal and tumour DNA from each patient was analysed using probe–enzyme combinations which identify restriction fragment length polymorphisms (RFLPs) in a large proportion of individuals. Normal DNA samples which were polymorphic at a given locus were considered 'informative', whereas homozygous samples were 'uninformative'. The signal intensity of fragments was determined by visual examination and confirmed by densitometry. The amount of paired normal and tumour DNA loaded onto the lanes (assessed with control probes on other chromosomes) was taken into account when judging the loss of allele in the tumour DNA. LOH was considered to occur when the intensity of the allele in the tumour DNA was less than 50% of that in corresponding normal tissue DNA. This partial loss is due either to contaminating normal tissue or to tumour heterogeneity.

### RNA analysis and quantification

RNA was extracted from normal and tumour tissue by using the lithium chloride/urea method (Auffray and Rougeon, 1980). Ten micrograms of RNA was fractionated by electrophoresis on 1.2% agarose gels containing 6% formaldehyde and analysed by blot hybridization after transfer onto nylon membrane filters (Hybond N, Amersham). The filters were hybridized with a nick-translated $^{32}$P-labelled human stathmin probe [1500-kb *SmaI–ClaI* fragment of plasmid p19.6 (Maucuer et al, 1990; Curmi et al, 1994)] in 50% formamide at 42°C. Membranes were washed in stringent conditions in 0.1 × SSPE (1 × SSPE: 150 mM sodium chloride, 9 mM sodium phosphate, 1 mM EDTA) and 0.1% SDS at 50°C and subjected to autoradiography at –80°C. Membranes were rehybridized with a 36B4 cDNA control probe [0.7-kb *Pst*I fragment as described (Masiakowski et al, 1982)] corresponding to a ubiquitous RNA. This control probe served in each experiment as an internal reference for the integrity of the RNA preparation and to normalize the amount of RNA loaded on the gel. The relative intensity of the mRNA bands was first assessed by visual examination and then by densitometry. Stathmin transcript levels in tumours were quantified relative to those in normal breast tissue by serial dilution of tumour RNA, until the Northern hybridization signals reached similar intensities. Stathmin transcript levels in tumours were scored as B (basal), M (moderate) or H (high).

### Immunohistochemistry (IHC)

Preliminary experiments on formalin- or Bouin-fixed tissue sections showed that this material was suitable for use with our anti-stathmin antiserum. Fixed sections were deparaffinized twice in xylene, rehydrated through a graded series of ethanols from 100% to 30% and then immersed in tap water. After three 10-min washes in phosphate-buffered saline (PBS)–glycine 0.1 M, non-specific binding was blocked by three 10-min incubations in PBS containing 3% bovine serum albumin (BSA). The primary anti-stathmin antiserum directed against peptide I of rat stathmin (Koppel et al, 1990) was applied at a 1:150 dilution to the slides and incubated overnight at 4°C in a moist chamber. After six 10-min washes with PBS–Tween 0.1%, fluorescein isothiocyanate (FITC)-conjugated goat anti-rabbit IgG antiserum (Tago, CA, USA) diluted 1:200 was applied to the slides, which were again incubated for 1 h at room temperature. After washing (nine 10-min washes with PBS–Tween 0.1%), slides were mounted with Mowiol (Mowiol 10%, glycerol 25%, Tris 100 mM). The preparations were observed with a conventional fluorescence microscope. A negative control was used for each tumour, with a 100 molar excess of antigen peptide during the staining procedure to

 © Cancer Research Campaign 1998

neutralize the primary antibody specific binding. Immunohistochemistry (IHC) results were analysed by two independent investigators, discordant results being reviewed together. For Ki-67 staining, sections were deparaffinized as described above, washed twice with PBS–glycine 100 mM, then boiled in citrate buffer pH 6 in a pressure cooker for 4 min. Sections were rinsed in PBS, and endogenous peroxidase activity was blocked by incubation for 15 min at room temperature in 0.3% (v/v) hydrogen peroxide in PBS. Sections were then washed three times in PBS and incubated with rabbit anti-human Ki-67 antigen at 1:50 dilution (Dako, Denmark). After three 5-min washes in PBS, the binding of the primary antibody was visualized with the Dako LSAB-2 kit. Finally, sections were counterstained with haematoxylin then mounted with Aquatex (Merk, France). The Ki-67 labelling index represents the percentage of positively stained nuclei, reported to the total number of tumour cells (at least 1000 cells by section) counted across photomicrographs of representative fields of the section.

### Protein analysis

### *Protein extraction and quantification*

Frozen biopsy specimens were available for protein extraction in a subset of seven breast tumours. Samples (6–53 mg) were sonicated twice for 60 s on ice in 500 µl of extraction buffer (20 mM Tris-HCl pH 8, 10 µg $ml^{-1}$ leupeptin, 25 µg $ml^{-1}$ aprotinin, 10 µg $ml^{-1}$ pepstatin, 1 mM EGTA). The disrupted tissues were centrifuged at 4°C and 100 000 r.p.m. for 6 min in a Beckman TL-100 centrifuge. Protein was assayed by the method of Bradford (1976) using BSA as standard.

### *Polyacrylamide gel electrophoresis*

One-dimensional electrophoresis was performed on 13% polyacrylamide gels (1D PAGE) (Laemmli, 1970).

Two-dimensional PAGE (2D PAGE) was performed according to Garrels (1979) with modifications (Sobel and Tashjian, 1983). Isoelectric focusing gels contained 2% total ampholines (Pharmacia, Sweden), pH 5–8 and 3.5–10 in the proportion 4:1 for the analysis of stathmin isoforms. The second dimension was run on 13% polyacrylamide gels. Proteins were either silver-stained on fixed gels as previously described, or immunoblotted (see below).

### *Quantification of total stathmin in tumours by Western blotting*

Preliminary 1D PAGE separation of equal amounts of tumour protein revealed that samples contained, in addition to cellular protein, variable amounts of plasma protein (the prominent visible variation concerend serum albumin). Use of the total protein content to determine and compare the stathmin content of tumours was thus inappropriate. Instead, we expressed stathmin content relative to the amount of actin (considered as an intracellular reference).

Cell proteins were separated by 13% sodium dodecyl sulfate polyacrylamide gel electrophoresis (SDS-PAGE) and transferred onto 0.2-µm nitrocellulose filters (Schleicher & Schuell, Germany) in a semi-dry electroblotting apparatus (transfer buffer: 48 mM Tris, 39 mM glycine, containing 20% isopropanol). The membrane was saturated with 5% non-fat dry milk in immunoblot solution (12 mM Tris-HCl pH 7.4, 160 mM sodium chloride, 0.1% Triton X-100) and probed with the same antiserum used for stathmin detection in IHC, but at a 1:10 000 dilution. A mouse monoclonal antibody against actin (N350, Amersham) was used at a 1:1000 dilution. Bound antibodies were detected with ECL (Amersham) and the filters were exposed to XAR5 film (Kodak, NY, USA).

The integral optical density of stathmin spots on trans-illuminated autoradiograms was measured with a BioProfil (Vilber Lourmat, France) image analysis system, after background treatment. Absolute quantification of stathmin protein was performed by comparing the integrated optical density of the stathmin band in tumour extracts with a standard scale constructed using recombinant protein (concentration assayed by amino acid analysis and measured simultaneously on the same film as experimental samples). Results with different exposure times differed by less than 10%. Results are expressed as absolute amounts of stathmin per arbitrary unit of actin.

### *Quantification of the relative amounts of stathmin isoforms*

Separation of stathmin isoforms was performed by 2D PAGE. The amounts loaded on the gels were equilibrated for the total amount of stathmin in each sample (determined by 1D Western blots). After separation, stathmin isoforms were revealed by Western blotting as described above. Stathmin isoforms were identified by comigrating paired tumour samples with a radiolabelled sample containing most of the known stathmin isoforms. The latter consisted of extracts from [$^{35}$S]methionine-labelled PC12 cells stimulated by nerve growth factor (NGF)/forskolin (2.5 S NGF, 200 ng $ml^{-1}$ overnight; forskolin 100 µM for 1 h) (Doye et al, 1990), after immunoprecipitation with an antistathmin antiserum.

Quantification was performed as described above. Results are expressed as the relative amount of stathmin isoforms in each tumour.

### Statistical analysis

Differences were analysed for statistical significance by using the chi-square test with Yate's correction to adjust for the continuity of the chi-squared distribution, when appropriate. Differences between the two populations were judged significant at a confidence level greater than 95% ($P < 0.05$).

**Figure 1** Total RNA extracted from breast specimen from women undergoing cosmetic breast surgery (N), adjacent normal tissue from breast cancer patients (P) and breast tumour tissue (T) was electrophoresed on agarose gels. Northern blotting was performed with the $^{32}$P-labelled stathmin probe. Rehybridization of the blot with the 36B4 probe demonstrated that comparable amounts of RNA were loaded in each case. The normal breast tissues (N and P) show a low level of stathmin mRNA (scored B). Amounts of stathmin mRNA in breast tumours varied from basal (scored B: T2, T3, T4 and T13) to moderate (scored M: T5 and T8) and high (scored H: T7)

© Cancer Research Campaign 1998

**Table 1** Comparison between LOH on chromosome 1p32–1pter and overexpression of stathmin mRNA

| Stathmin expression | 1p32–1pter LOH Yes | No | $P^a$ |
|---|---|---|---|
| Basal | 13 | 17 | NS |
| Overexpressing | 8 | 2 | $P = 0.082$ |

$^a\chi^2$ test; NS, not significant.

A

B

| Sample | Stathmin (normalized units) | mRNA score |
|---|---|---|
| Normal breast tissue | 1.0 | B |
| Tumour T1 | 0.7 | B |
| T2 | 0.9 | B |
| T3 | 1.3 | B |
| T5 | 9.4 | M |
| T6 | 13.4 | M |
| T7 | 76.6 | – H |

**Figure 2** (**A**) Western blot analysis of stathmin in human breast tumours. Whole cellular proteins were prepared from frozen biopsies, separated on SDS-PAGE, electroblotted on nitrocellulose membrane and reacted with anti-stathmin antiserum and anti-actin monoclonal antibodies as described under 'Patients and methods'. Tumours T1, T2 and T3 (scored B by Northern blotting) expressed basal levels of stathmin. Tumours T4 and T5 (scored M by Northern blotting) expressed moderate levels, whereas tumour T7 (scored H by Northern blotting) expressed high levels of stathmin. (**B**) Normalized expression of stathmin. Normalized expression represents the ratio of stathmin to actin in each tumour to the mean ratio of stathmin to actin in normal breast tissue. Three groups of tumours were identified matching the groups based on stathmin mRNA scoring: (i) tumours T1, T2 and T3 expressed basal levels of stathmin (basal ratio), (ii) tumours T5 and T6 showed moderate overexpression (ratio of stathmin to actin around 10 times the basal ratio) and (iii) tumour T7 expressed a high level of stathmin (stathmin/actin ratio about 75 times above the basal ratio)

### RESULTS

### Stathmin mRNA expression in normal breast tissue

RNA was extracted from normal breast specimens obtained from eight women undergoing cosmetic breast surgery and six breast cancer patients. Northern blot hybridization revealed a clear stathmin signal with a normal size of 1 kb, which was normalized to the 36B4 control signal. Stathmin mRNA expression was weak in all the control specimens and was scored as basal (B) (Figure 1).

### Stathmin mRNA overexpression in one-third of breast tumours

Northern blotting of the 50 human breast carcinomas showed that all expressed a stathmin mRNA of the normal size. However, major differences in the amount of stathmin messenger were observed: 35 tumours scored B (basal), ten gave a signal 3–5 times that of normal breast tissue (scored M) and five gave a greater than sixfold stronger signal (scored H) (Figure 1). The strongest expression was 32-fold the basal level in tumour no. T7.

### Correlation between stathmin mRNA overexpression and clinical and pathological parameters

Overexpression of stathmin mRNA was not significantly associated ($\chi^2$ analysis) with standard prognostic feature including macroscopic tumour size, histopathological grade, lymph node or steroid receptor status (data not shown) and with the Ki-67 labelling index. The last feature showed great variations from 4.7% to 21.2% in tumours scored B, and from 1.9% to 18.5% in stathmin-overexpressing tumours.

### Tentative link between stathmin mRNA levels and 1p32–1pter LOH

Southern blots of restricted genomic DNA with the stathmin cDNA probe (which is not polymorphic) showed no rearrangement or amplification of the stathmin gene in the overexpressing tumours compared with the controls in any of the 50 human breast tumours tested (data not shown).

Forty of the 50 tumours have also been tested for LOH with five polymorphic DNA markers (D1S80, D1S76, D1S7, D1S57 and MYCL1) located in the 1p32–pter region: 21 (52.5%) showed LOH on the telomeric region, while the remainder had a normal DNA profile. As shown in Table 1, we found a trend towards a link between 1p32–pter LOH and stathmin mRNA overexpression ($P = 0.082$). Indeed, 80% (8/10) of the tumours overexpressing the stathmin gene showed deletions in the 1p32–pter region, compared with 43% (13/30) of the tumours expressing basal levels of stathmin mRNA.

### High stathmin protein expression in tumours with high stathmin mRNA levels

Stathmin protein expression was investigated in seven tumours for which frozen samples were available. Four of these tumours scored B for stathmin mRNA, two scored M and one scored H. We first evaluated the stathmin content in each of these tumours by Western blotting of 5 μg of high-speed centrifugation supernatant protein. A parallel silver-stained gel, run with the same amount of sample, revealed various degrees of contamination by serum protein. Blots were thus probed with an anti-actin antibody to normalize the amount of loaded tissue protein to this cellular protein marker. As shown in Figure 2A, tumours scoring M and H by Northern blotting contained far larger amounts of stathmin than tumours scoring B; tumours scoring M expressed about ten times

© Cancer Research Campaign 1998

**Figure 3** Two-dimensional analysis of stathmin in tumours expressing basal (row B) or moderate and high levels of stathmin (row M/H) as explained in 'Patients and methods'. A protein extract from $^{35}$S-methionine-labelled PC12 cells stimulated by NGF/forskolin was used as control. Stathmin was found essentially unphosphorylated in all the tumours, migrating as the N isoform observed in the control specimen. A small amount of stathmin was phosphorylated in about half the tumours, with no pattern specific to the M/H subgroup of tumours. The minor spot that appears adjacent to P1 (left side) for T1, 2, 3, 5 and 7 is known as the minor β-isoform, an unphosphorylated post-translational variant of stathmin (Beretta et al, 1989*b*)

more stathmin than tumours scoring B. The strongest expression was about 75 times that in tumours scoring B; this tumour (no. T7) also contained the highest stathmin mRNA level (Figure 2B).

### Stathmin phosphorylation is not related to stathmin expression levels

Stathmin phosphorylation was examined on Western blots after separation of stathmin isoforms by 2D PAGE. A reference PC12 cell sample containing the radiolabelled stathmin isoforms N (major non-phosphorylated form), P1 (stathmin isoform phosphorylated at one site) and P2 (stathmin isoform phosphorylated at two sites) (Beretta et al, 1989*b*) was co-migrated with the tumour samples to identify the observed isoforms. Stathmin in tumours was mainly present as its unphosphorylated isoform N. A variable amount of protein was phosphorylated and migrated to position P1. The P1/N ratio ranged from 0.08 to 0.3 (for comparison, the P1/N ratio was observed between 0.1 and 0.15 in PC12 cells) (Doye et al, 1990). No pattern of stathmin phosphorylation specific to stathmin-overexpressing tumours was identified (Figure 3).

### Localization of stathmin overexpression to tumour cell cytoplasm

Of the 14 tumours studied by IHC, we detected specific immunoreactivity in the five tumours which overexpressed stathmin mRNA. Interestingly, the bulk of stathmin immunoreactivity was found inside tumour cells (Figure 4), whereas infiltrating lymphocytes were found weakly positive or devoid of stathmin immunoreactivity. Normal glandular cells isolated in the tumour or in the normal parenchyma were also unlabelled. Tumour cell labelling was found exclusively in the cytoplasm, and the staining pattern consisted either of isolated positive cells or diffuse positivity throughout the section. Stathmin was never overexpressed in tumours scoring B by Northern blotting. We thus observed a perfect match between stathmin mRNA overexpression, high levels of stathmin by Western blotting and IHC positivity (Table 2).

### DISCUSSION

Interest in the study of stathmin in tumours grew with the observation that stathmin, a 19-kDa cytosolic phosphoprotein, is subject to marked variations in its expression and phosphorylation pattern during cell regulation, triggered by signals as diverse as hormones, growth factors and differentiation factors (Strahler et al, 1992; Larsson et al, 1995). Recently, attention was raised when it was discovered that stathmin is a major microtubule-destabilizing factor in vitro (Belmont and Mitchison, 1996) and in vivo. Both the overexpression of wild type stathmin or of a cdk target site mutant elicited rapid depolymerization of tubulin (Marklund et al, 1996). It was also noticed that the overexpression of a non-phosphorylatable mutant of stathmin resulted in a large population of cells blocked at G2/M with a high DNA content (Marklund et al, 1994*b*; Larsson et al, 1995; Lawler et al, 1997). To account for these effects, we demonstrated that stathmin directly interacts with, and sequesters, tubulin (Curmi et al, 1997) in a $T_2S$ complex (Jourdain et al, 1997), and that this sequestration leads to the displacement of the microtubule/tubulin equilibrium towards depolymerization of microtubules (Jourdain et al, 1997). Importantly, phosphorylation of stathmin altered the affinity of stathmin for tubulin (Marklund et al, 1996; Curmi et al, 1997; Di Paolo et al, 1997; Horwitz et al, 1997; Larsson et al, 1997). Together, these results give insight into the observed physiological variations of stathmin phosphorylation during the mitotic cycle (Strahler et al, 1992; Brattsand et al, 1994) and argue for the search for stathmin dysregulations in tumours as well as an understanding of its mechanisms.

In the present study, using genomic DNA, mRNA and protein analysis, we assessed, for the first time, stathmin expression in human breast cancer. The main finding was that there is a strong expression of stathmin mRNA and protein in one-third of the tumours examined. Among the 50 breast tumours studied, 15 overexpressed stathmin mRNA (3–32 times basal values), with protein levels ranging from 10 to 70 times the basal value. This overexpression, also assessed by immunohistochemistry, was contributed almost exclusively by cancer cells, with immunoreactivity localized exclusively in the cytoplasm. The results of these three methods is in good agreement, as a correlation is observed between high levels of stathmin mRNA and high stathmin protein content. This indicates that any of the three methods used here could be employed as a screening tool for larger studies. Stathmin, in overexpressing tumours, is found mainly in its unphosphorylated form N, the potentially active form for its interaction with tubulin. This finding, which may have an important pathophysiological significance, is presently under investigation.

There have been conflicting data concerning stathmin overexpression in malignant processes. Our results show that stathmin overexpression in breast cancer is not a constant feature, a trait already found in other hormone-dependent cancers, such as prostate adenocarcinomas (Friedrich et al, 1995), and in neuroblastomas (Hailat et al, 1990). On the other hand, stathmin is found to be more abundant in acute leukaemias of different lineages than in non-leukaemic cells (Hanash et al, 1988). For breast cancer, stathmin overexpression may thus delineate a new subgroup of tumours.

With regard to the possible link between stathmin overexpression and tumour cell proliferative potential, we measured the Ki-67 labelling index in tumours and we were not able to correlate this with stathmin expression. This latter observation might be

© Cancer Research Campaign 1998

**Figure 4** Two breast invasive carcinomas scored H and considered as diffusely positive (**A**, **C**, **E** and **B**, **D**, **F** respectively) are presented after haematoxylin–eosin and saffron staining (**A**,**B**), and immunohistochemical detection of stathmin (**C**,**D**). The peptide absorbed anti-stathmin antiserum was used as a control (**E**,**F**) (bar = 40 μm). The tumour on the left displays a massive and trabecular pattern of tumour proliferation (**A**). Immunohistochemical detection of stathmin (**C**) shows positive cytoplasmic staining in the breast carcinoma cells, the tumour cell nuclei remaining unstained (arrow). The negative control (**E**) shows no reactivity in the previously immunostained tumour cells. The tumour on the right is from a carcinoma with isolated tumour cells or nests of tumour cells in a stroma rich in lymphocytes (**B**). Immunohistochemical detection of stathmin (**D**) is found in the cytoplasm of tumour cells (arrow). The negative control (**F**) shows no reactivity in the previously immunostained tumour cells because of the usually large variations found with this marker (Bouzubar et al, 1989). Studies on larger series will answer this question. Data relating stathmin expression and the proliferation potential of tumours reported in the literature are, apparently, somewhat confusing. In natural conditions, up-regulation of stathmin has been found to be neither uncoupled from cell proliferation nor restricted to cell types with proliferative potential (Brattsand et al, 1993). In non-Hodgkin's lymphoma and in Hodgkin's disease, Hodgkin and Reed-Sternberg cells frequently express stathmin with strong staining intensity, but stathmin overexpression is only partly related to cell proliferation (Nylander et al, 1995). In contrast, stathmin transfection into lymphoblastoid cells results in a partial inhibition of cell proliferation (Brattsand et al, 1993), and antisense transfection into leukaemic cells reverses the malignant phenotype (Jeha et al, 1996).

To interpret these observations, one must consider that stathmin is at the heart of a complex signalling network, being a direct substrate for different kinases: the MAP kinase family (Leighton

 © Cancer Research Campaign 1998

**Table 2** Summary of the results for stathmin expression and phosphorylation

| Tumour no. | Northern | WB | IHC | 2DWB |
|---|---|---|---|---|
| T1 | B | 1 | ud | N |
| T2 | B | 1 | ud | NP |
| T3 | B | 1 | ud | NP |
| T4 | B | 1 | ud | N |
| T5 | M | 9 | + | N |
| T6 | M | 13 | + | N |
| T7 | H | 77 | + | NP |
| T8 | M | ND | + | ND |
| T9 | M | ND | + | ND |
| T10 | B | ND | UD | ND |
| T11 | B | ND | UD | ND |
| T12 | B | ND | UD | ND |
| T13 | B | ND | UD | ND |
| T14 | B | ND | UD | ND |

By Northern blotting, tumours were scored basal (B), moderate (M) or high (H). Western blotting (WB) showed basal levels of stathmin (1) or increased levels (ranging from 10 to 77 times the basal value; values are expressed as defined in Figure 2). Immunohistochemical studies (IHC) showed tumours with no stathmin reactivity (ud) or specific stathmin labelling in tumour cells (+). Note that a perfect match was found for stathmin expression at the mRNA and protein levels (Northern, WB and IHC). Stathmin phosphorylation was examined after separation of stathmin isoforms by 2D-PAGE as described in Figure 3. No specific pattern of stathmin phosphorylation was observed for basal or overexpressing tumours. N, stathmin essentially in its unphosphorylated state; NP, stathmin displaying a significant proportion of its P1 phosphorylated form in addition to its unphosphorylated state. B, basal; UD, undetectable; N, $0.08 < P/N < 0.15$; M, moderate; ND, not determined; NP, $0.15 < P/N < 0.30$. H, high et al, 1993), cAMP-dependent protein kinase (Beretta et al, 1993), p34cdc2 kinase (Beretta et al, 1993; Brattsand et al, 1994; Larsson et al, 1995) and the $Ca^{2+}$–calmodulin-dependent kinases II and IV (Marklund et al, 1994*a*; Le Gouvello et al, 1998). Furthermore, stathmin interacts with various protein partners, for which we have identified several candidates (Maucuer et al, 1995). One of these, CC2/tsg101, interestingly being the product of a tumour suceptibility gene (Li and Cohen, 1996), was suggested to be implicated in breast cancer (Li et al, 1997). The intricate regulation of stathmin and of its partners being highly probable, we speculate that stathmin overexpression might contribute to tumorigenesis in different ways.

1. it could represent a normal reaction to cell proliferation itself. In fact, a recent study in our laboratory showed that a high cell density in culture induces stathmin expression, most likely triggered by cell–cell contacts. Stathmin expression, in that case, is likely being up-regulated, in relation to the limitation of cell overgrowth at the stage preceding cell differentiation (Balogh et al, 1996). This cell culture result is in good agreement with the induction of stathmin expression during liver regeneration, stathmin displaying a delayed expression peak following the mitotic peak and correlating with the slowdown in cell proliferation (Koppel et al, 1993). Stimulated expression of stathmin may thus be part of a regulatory programme aimed at limiting cell overproliferation, and also activated, although inefficiently, in transformed tumoral cells.
2. Alternatively, overexpression of stathmin might reflect an alteration of stathmin itself, leading to the malignant phenotype; mutations in the structural gene that are undetectable by blotting techniques would then remain to be identified.
3. Finally, cells might react to changes in stathmin protein partners (Maucuer et al, 1995) in a feedback pathway.

The other interesting finding in this study is the tentative link between loss of heterozygosity in the 1p32–1pter region and stathmin overexpression. Deletions of the short arm of chromosome 1, especially the telomeric 1p32–pter region, have been detected by both molecular and cytogenetic approaches in breast tumours, suggesting that this region contains a breast tumour-suppressor gene (Bièche et al, 1994). Interestingly, this region also houses the stathmin gene (mapping to 1p35–36.1). Stathmin appears thus to be a good candidate for being one of the tumour suppressor genes located in this chromosome region. The finding of a tentative link between LOH in the 1p32–1pter region and stathmin overexpression may appear surprising, but it is reminiscent of the coexistence of *p53* gene LOH and overexpression of the corresponding protein. In this case, mutations were found either in the regulatory or in the coding region of the *p53* gene (Aka et al, 1993; Ohgaki et al, 1993; Greenblatt et al, 1994). Similar mutations may have occurred in the vicinity of or within the stathmin gene. Alternatively, DNA removal may have brought a powerful enhancer close to the stathmin gene to account for the increase in mRNA levels.

In conclusion, our study has clearly established that a significant proportion of breast cancers overexpress stathmin and may define a new breast cancer subtype. Further studies with a larger population and longer follow-up will allow the evaluation of the prognostic significance of stathmin overexpression, as well as an exploration of the status of the stathmin protein partners in the overexpressing tumours.

### ACKNOWLEDGEMENTS

We are indebted to Dr A Maucuer for the human stathmin probe and to Dr P Chambon for probe 36B4. We also thank Dr D Rickman for critical reading of this manuscript. This work was supported by the Ligue Nationale Contre le Cancer (LNCC), the Association pour la Recherche contre le Cancer (ARC) and the Institut National de la Santé et de la Recherche Médicale (INSERM).

### REFERENCES

Aka K, Brunner JM, Bondy ML, Ligon K, Nishi T, del Giglio A, Moser RP, Levin VA and Saya H (1993) Detection of p53 alterations in human astrocytomas using frozen tissue sections for the polymerase chain reaction. *J Neurooncol* **16**: 125–133

Auffray C and Rougeon F (1980) Purification of mouse immunoglobulin heavy-chain messenger RNAs from total myeloma tumor RNA. *Eur J Biochem* **107**: 303–314

Balogh A, Mege RM and Sobel A (1996) Cell density dependent expression of stathmin in C2 myoblasts in culture. *Exp Cell Res* **224**: 8–15

Belmont LD and Mitchison TJ (1996) Identification of a protein that interacts with tubulin dimers and increases the catastrophe rate of microtubules. *Cell* **84**: 623–631

Beretta L, Boutterin MC and Sobel A (1988) Phosphorylation of intracellular proteins related to the multihormonal regulation of prolactin: comparison of normal anterior pituitary cells in culture with the tumor-derived GH cell lines. *Endocrinology* **122**: 40–51

Beretta L, Boutterin MC, Drouva S and Sobel A (1989*a*) Phosphorylation of a group of proteins related to the physiological, multihormonal regulations of the various cell types in the anterior pituitary gland. *Endocrinology* **125**: 1358–1364

Beretta L, Houdouin F and Sobel A (1989*b*) Identification of two distinct isoforms of stathmin and characterization of their respective phosphorylated forms. *J Biol Chem* **264**: 9932–9938

Beretta L, Dobransky T and Sobel A (1993) Multiple phosphorylation of stathmin: identification of four sites phosphorylated in intact cells, and in vitro by cyclic-AMP dependent protein kinase and p34cdc2. *J Biol Chem* **268**: 20076–20084

Bièche I, Champème MH and Lidereau R (1994) A tumor suppressor gene on chromosome 1p32–pter controls the amplification of MYC family genes in breast cancer. *Cancer Res* **54**: 4274–4276

Bloom HJG and Richardson WW (1957) Histological grading and prognosis in breast cancer. *Br J Cancer* **11**: 359–377

Bouzubar N, Walker KJ, Griffiths K, Ellis IO, Elston CW, Robertson JFR, Blamey RW and Nicholson RI (1989) Ki67 immunostaining in primary breast cancer: pathological and clinical associations. *Br J Cancer* **59**: 943–947

Bradford M (1976) A rapid and sensitive method for the quantification of microgram quantities of protein, utilizing the principle of protein-dye binding. *Anal Biochem* **72**: 248–254

Brattsand G, Roos G, Marklund U, Ueda H, Landberg G, Nanberg E, Sideras P and Gullberg M (1993) Quantitative analysis of the expression and regulation of an activation-regulated phosphoprotein (oncoprotein 18) in normal and neoplastic cells. *Leukemia* **7**: 569–579

Brattsand G, Marklund U, Nylander K, Roos G and Gullberg M (1994) Cell-cycle-regulated phosphorylation of oncoprotein 18 on ser16, ser25, and ser38. *Eur J Biochem* **220**: 359–368

Braverman R, Bhattacharya B, Feuerstein N and Cooper HL (1986) Identification and characterization of the nonphosphorylated precursor of pp17, a phosphoprotein associated with phorbol ester induction of growth arrest and monocytic differentiation in HL-60 promyelocytic leukemia cells. *J Biol Chem* **261**: 14342–14348

Chneiweiss H, Cordier J and Sobel A (1992) Stathmin phosphorylation is regulated in striatal neurons by vasoactive intestinal peptide and monoamines via multiple intracellular pathways. *J Neurochem* **58**: 282–289

Cooper HL, McDuffie E and Braverman R (1989) Human peripheral lymphocyte growth regulation and response to phorbol esters is linked to synthesis and phosphorylation of the cytosolic protein, prosolin. *J Immunol* **143**: 956–963

Cooper HL, Fuldner R, McDuffie E and Braverman R (1990) A specific defect of prosolin phosphorylation in T-cell leukemic lymphoblasts is associated with impaired down-regulation of DNA synthesis. *J Immunol* **145**: 1205–1213

Curmi P, Maucuer A, Asselin S, Lecourtois M, Chaffotte A, Schmitter JM and Sobel A (1994) Molecular characterization of human stathmin expressed in *Escherichia coli*: site-directed mutagenesis of two phosphorylatable serines (Ser-25 and Ser-63) *Biochem J* **300**: 331–338

Curmi PA, Andersen SSL, Lachkar S, Gavet O, Karsenti E, Knossow M and Sobel A (1997) The stathmin tubulin interaction in vitro. *J Biol Chem* **272**: 25029–25036

Di Paolo G, Pellier V, Catsicas M, Antonsson B, Catsicas S and Grenningloh G (1996) The phosphoprotein stathmin is essential for nerve growth factor-stimulated differentiation. *J Cell Biol* **133**: 1383–1390

Di Paolo G, Antonsson B, Kassel D, Riederer BM and Grenningloh G (1997) Phosphorylation regulates the microtubule-destabilizing activity of stathmin and its interaction with tubulin *FEBS Lett* **416**: 149–152

Doye V, Boutterin MC and Sobel A (1990) Phosphorylation of stathmin and other proteins related to nerve growth factor-induced regulation of PC12 cells. *J Biol Chem* **265**: 11650–11655

Doye V, Kellermann O, Buc-Caron MH and Sobel A (1992) High expression of stathmin in multipotential teratocarcinoma and normal embryonic cells versus their early differentiated derivatives. *Differentiation* **50**: 89–96

EORTC Breast Cooperative Group Revision (1980) Revision of the standards for the assessment of hormone receptors in human breast cancer. Report of the second EORTC workshop. *Eur J Cancer* **16**: 1513–1515

Ferrari AC, Seuanez HN, Hanash SM and Atweh GF (1990) A gene that encodes for a leukemia-associated phosphoprotein (p18) maps to chromosome bands 1p35-36.1. *Genes Chrom Cancer* **2**: 125–129

Friedrich B, Grönberg H, Landström M, Bergh A and Gullberg M (1995) Differentiation-stage specific expression of oncoprotein 18 in human and rat prostatic adenocarcinoma. *Prostate* **27**: 102–109

Garrels JI (1979) Two-dimensional gel electrophoresis and computer analysis of proteins synthesized by clonal cell lines. *J Biol Chem* **254**: 7961–7977

Ghosh PK, Anderson J, Cohen N, Takeshita K, Atweh GF and Lebowitz P (1993) Expression of the leukemia-associated gene, p18, in normal and malignant tissues; inactivation of expression in a patient with cleaved B-cell lymphoma/leukemia. *Oncogene* **8**: 2869–2872

Greenblatt MS, Bennet WP, Hollstein M and Harris CC (1994) Mutations in the p53 tumor suppressor gene: clues to cancer etiology and molecular pathogenesis. *Cancer Res* **54**: 4855–4878

Hailat N, Strahler JR, Melhem RF, Zhu XX, Brodeur G, Seeger RC, Reynolds CP and Hanash SM (1990) N-myc gene amplification in neuroblastoma is associated with altered phosphorylation of a proliferation related polypeptide (Op 18). *Oncogene* **5**:1615–1618

Hanash SM, Strahler JR, Kuick R, Chu EHY and Nichols D (1988) Identification of a polypeptide associated with the malignant phenotype in the acute leukemia. *J Biol Chem* **263**: 12813–12815

Horwitz SB, Shen H, He L, Dittmar P, Neef R, Chen J and Schubart UK (1997) The microtubule-destabilizing activity of metablastin (p19) is controlled by phosphorylation. *J Biol Chem* **272**: 8129–8132

Jeha S, Luo X, Beran M, Kantarjian H and Atweh GF (1996) Antisense RNA inhibition of phosphoprotein p18 expression abrogates the transformed phenotype of leukemic cells. *Cancer Res* **56**: 1445–1450

Jourdain L, Curmi P, Sobel A, Pantaloni D and Carlier MF (1997) Stathmin is a tubulin-sequestering protein which forms a ternary T2S complex with two tubulin molecules. *Biochemistry* **36**: 10817–10821

Koppel J, Boutterin MC, Doye V, Peyro-Saint-Paul H and Sobel A (1990) Developmental tissue expression and phylogenetic conservation of stathmin, a phosphoprotein associated with cell regulations. *J Biol Chem* **265**: 3703–3707

Koppel J, Loyer P, Maucuer A, Rehák P, Manceau V, Guguen-Guillouzo C and Sobel A (1993) Induction of stathmin expression during liver regeneration. *FEBS Lett* **331**: 65–70

Laemmli UK (1970) Cleavage of structural proteins during assembly of the head of bacteriophage T4. *Nature* **227**: 680–685

Larsson N, Melander H, Marklund U, Osterman O and Gullberg M (1995) G2/M transition requires multisite phosphorylation of oncoprotein 18 by two distinct protein kinase systems. *J Biol Chem* **270**: 14175–14183

Larsson N, Marklund U, Gradin HM, Brattsand G and Gullberg M (1997) Control of microtubule dynamics by oncoprotein 18: dissection of the regulatory role of multisite phosphorylation during mitosis. *Mol Cell Biol* **17**: 5530–5539

Lawler S, Gavet O, Rich T and Sobel A (1998) Stathmin overexpression in 293 cells affects signal transduction and the cell cycle. *FEBS Lett* **421**: 55–60

Le Gouvello, Manceau V and Sobel A (1998) Serine 16 of stathmin as a cytosolic target for $Ca^{2+}$/Calmodulin-dependent kinase II after CD2 triggering of human T lymphocytes. *J Immunol* **161**: (in press)

Leighton I, Curmi P, Campbell DG, Cohen P and Sobel A (1993) The phosphorylation of stathmin by MAP kinase. *Mol Cell Biochem* **127/128**: 151–156

Li L and Cohen SN (1996) *tsg101:* a novel tumor susceptibility gene isolated by controlled homozygous functional knockout of allelic loci in mammalian cells. *Cell* **85**: 319–329

Li L, Li X, Francke U and Cohen SN (1997) The tsg101 tumor suceptibility gene is located in chromosome 11 band p15 and is mutated in human breast cancer. *Cell* **88**: 143–154

Luo X, Mookerjee B, Ferrari A, Mistry S and Atweh GF (1994) Regulation of phosphoprotein p18 in leukemic cells. Cell cycle regulated phosphorylation by $p34^{cdc2}$ kinase. *J Biol Chem* **269**: 10312–10318

Marklund U, Larsson N, Brattsand G, Osterman O, Chatila TA and Gullberg M (1994*a*) Serine 16 of oncoprotein 18 is a major cytosolic target for the $Ca^{2+}$/calmodulin-dependent kinase-Gr. *Eur J Biochem* **225**: 53–60

Marklund U, Osterman O, Melander H, Bergh A and Gullberg M (1994*b*) The phenotype of a 'cdc2 kinase target site-deficient' mutant of oncoprotein 18 reveals a role of this protein in cell cycle control. *J Biol Chem* **269**: 30626–30635

Marklund U, Larsson N, Melander Gradin H, Brattsand G and Gullberg M (1996) Oncoprotein 18 is a phosphorylation-responsive regulator of microtubule dynamics. *EMBO J* **15**: 5290–5298

Masiakowski P, Breathnach R, Bloch J, Gannon F, Krust A and Chambon P (1982) Cloning of cDNA sequences of hormones-regulated genes from the MCF-7 human breast-cancer cell line. *Nucl Acids Res* **10**: 7895–7899

Maucuer A, Doye V and Sobel A (1990) A single amino acid difference distinguishes the human and the rat sequences of stathmin, a ubiquitous intracellular phosphoprotein associated with cell regulations. *FEBS Lett* **264**: 275–278

Maucuer A, Camonis JH and Sobel A (1995) Stathmin interaction with a novel putative kinase and coiled-coil forming protein domains. *Proc Natl Acad Sci USA* **92**: 3100–3104

Nylander K, Marklund U, Brattsand G, Gullberg M and Roos G (1995) Immunohistochemical detection of oncoprotein 18 (Op18) in malignant lymphomas. *Histochem J* **27**: 155–160

Ohgaki H, Eibl RH, Schwab M, Reichel MB, Mariani L, Gehring M, Petersen I, Höll T, Wiestler OD and Kleihues P (1993) Mutations of the p53 tumor suppressor gene in neoplasms of the human nervous system. *Mol Carcinog* **8**: 74–80

© Cancer Research Campaign 1998

Pasmantier R, Danoff A, Fleischer N and Schubart UK (1986) P19, a hormonally regulated phosphoprotein of peptide-hormone producing cells: secretagogue-induced phosphorylation in AtT-20 mouse pituitary tumor cells and in rat and hamster insulinoma cells. *Endocrinology* **19**: 1229–1238

Peyron J, Aussel C, Ferrua B, Häring H and Fehlmann M (1989) Phosphorylation of two cytosolic proteins. An early event of T-cell activation. *Biochem J* **258**: 505–510

Sambrook J, Fritsch EF and Maniatis T (1989) *Molecular Cloning: A Laboratory Manual*, 2nd edn. Cold Spring Harbor Laboratory: Cold Spring Harbor, NY

Sobel A (1991) Stathmin: a relay phosphoprotein for multiple signal transduction? *Trends Biochem Sci* **16**: 301–305

Sobel A and Tashjian Jr AH (1983) Distinct patterns of cytoplasmic protein phosphorylation related to regulation of synthesis and release of prolactin by GH cells. *J Biol Chem* **258**: 10312–10324

Sobel A, Boutterin MC, Beretta L, Chneiweiss H, Doye V and Peyro-Saint-Paul H (1989) Intracellular substrates for extracellular signaling: characterization of a ubiquitous, neuron-enriched phosphoprotein (stathmin). *J Biol Chem* **264**: 3765–3772

Strahler JR, Lamb BJ, Ungar DR, Fox DA and Hanash SM (1992) Cell cycle progression is associated with distinct patterns of phosphorylation of Op18. *Biochem Biophys Res Commun* **185**: 197–203

© Cancer Research Campaign 1998